# Correlation between gait parameters and clinical scales in individuals with Parkinson’s disease

**DOI:** 10.1590/1980-5764-DN-2024-0205

**Published:** 2025-09-05

**Authors:** Adriana Tanigawa Bitiati, Marina Ortega Golin, Daniel Boari Coelho

**Affiliations:** 1 Centro Universitário FMABC, Graduação Fisioterapia, Santo André SP, Brazil; 2 Universidade Federal do ABC, Centro de Matemática, Computação e Cognição, São Bernardo do Campo SP, Brazil.; 3 Universidade Federal do ABC, Engenharia Biomédica, São Bernardo do Campo SP, Brazil.

**Keywords:** Physical Therapy Modalities, Postural Balance, Biomechanical Phenomena, Movement Disorders, Modalidades de Fisioterapia, Equilíbrio Postural, Fenômenos Biomecânicos, Transtornos dos Movimentos

## Abstract

The ability to walk independently is essential for the quality of life of individuals with Parkinson’s disease (PD), and aspects such as mobility, balance, and cognition are important for walking. However, the association between these factors has not yet been fully established.

**Objective:**

To verify the correlations between cognitive and motor clinical scales, and gait intervals in individuals with PD.

**Methods:**

Twenty-two individuals with a clinical diagnosis of idiopathic PD were evaluated using balance and cognitive tests to classify the severity of the disease and gait analysis in the laboratory.

**Results:**

The Unified Parkinson’s Disease Rating Scale, motor part (UPDRS-III) was directly related to step width in the OFF state of medication (p=0.029); the Mini-Test of Balance Assessment System (Mini-BESTest) was inversely proportional to step width in medication ON (p=0.001) and OFF (p=0.005) states; Stroop-III was directly proportional to the stride time (p=0.039) and the stance (p=0.003) and double stance (p=0.011) phases and inversely proportional to the cadence (p=0.033) and swing phase (p=0.003), both in the ON state of medication; the Trail Making Test Part A (TMTB-A) was inversely proportional to step size (p=0.035), stride size (p=0.037), cadence (p=0.036) and speed (p=0.003) in the ON state.

**Conclusion:**

Cognitive dysfunctions are directly related to gait instability in PD, reinforcing the importance of interventions that integrate motor and cognitive rehabilitation. These strategies can improve gait safety and efficacy in individuals with PD.

## RESUMO

A habilidade de andar de maneira independente é essencial para a qualidade de vida em indivíduos com doença de Parkinson (DP), e aspectos como mobilidade, equilíbrio e cognição são importantes para a execução da marcha. Entretanto, ainda não está plenamente estabelecida a associação entre esses fatores.

**Objetivo:**

Verificar as correlações entre escalas clínicas cognitivas e motoras e parâmetros da marcha em indivíduos com DP.

**Métodos:**

Foram avaliados 22 indivíduos com diagnóstico clínico de DP idiopática por meio de testes de equilíbrio, cognitivos e testes para classificar a severidade da doença, além da análise de marcha no laboratório.

**Resultados:**

A *Unified Parkinson’s Disease Rating Scale, motor part* (UPDRS-III) se relacionou de maneira diretamente proporcional com a largura do passo no estado OFF da medicação (p=0,029); o *Mini-Test of Balance Assessment System* (Mini-BESTest) foi inversamente proporcional à largura do passo nos estados ON (p=0,001) e OFF (p=0,005) de medicação; o teste de Stroop-III se mostrou diretamente proporcional ao tempo da passada (p=0,039) e às fases de apoio (p=0,003) e de duplo apoio (p=0,011) e inversamente proporcional à cadência (p=0,033) e à fase de balanço (p=0,003), ambos no estado ON da medicação; o *Trail Making Test Part A* (TMTB-A) foi inversamente proporcional ao tamanho do passo (p=0,035), tamanho da passada (p=0,037), cadência (p=0,036) e velocidade (p=0,003) no estado ON.

**Conclusão:**

As disfunções cognitivas estão diretamente relacionadas à instabilidade da marcha na DP, reforçando a importância de intervenções que integrem reabilitação motora e cognitiva. Essas estratégias podem melhorar a segurança e a eficácia da marcha em indivíduos com DP

## INTRODUCTION

 Parkinson’s disease (PD) is primarily idiopathic, with aging as one of its key risk factors. As global life expectancy rises and the elderly population grows, understanding PD becomes increasingly critical^1^. The neuropathology of PD is marked by the degeneration of dopaminergic neurons in the substantia nigra and the accumulation of Lewy bodies due to alpha-synuclein deposition^2^. Neuronal loss in the substantia nigra disrupts dopaminergic modulation within the basal ganglia, causing an imbalance in the cortical-striatal-thalamic circuits, which are crucial for motor control^3^. This imbalance manifests as PD’s motor symptoms: bradykinesia, muscle rigidity, tremor, postural instability, and gait dysfunctions^4^. In PD, anticipatory postural adjustments are diminished, leading to disorganized muscle activation patterns that hinder voluntary movement^4^. This results in short, repetitive steps and challenges in initiating movements, such as turning or taking the first step, a phenomenon known as freezing of gait (FOG)^3^. 

 Diagnosis relies on clinical assessments, primarily the Unified Parkinson’s Disease Rating Scale (UPDRS), which evaluates disease severity across four domains: mental function, behavior, daily living activities, motor examination, and treatment complications^5^. The UPDRS provides a comprehensive measure of disability and disease progression in both medicated (ON state) and unmedicated (OFF state) conditions. 

 Cognitive decline is another common feature of PD, and it is evaluated using tools such as the Montreal Cognitive Assessment (MoCA). This brief test assesses various cognitive domains, with lower scores indicating more significant deficits^6^. Additionally, the Stroop Test measures selective attention by challenging individuals to name colors while suppressing the automatic tendency to read words^7^. Similarly, the Trail Making Test (TMT) evaluates cognitive flexibility and attention, with longer completion times reflecting worse performance^8,9^

 Balance impairments in PD arise from reduced postural adjustments, increasing the risk of falls and complicating gait automation^4^. The Mini-BESTest, a dynamic balance assessment, evaluates anticipatory postural adjustments, postural responses, sensory orientation, and dynamic gait stability^10^. Complementarily, the Falls Efficacy Scale-International (FES-I) quantifies perceived fall risk, with higher scores indicating a more significant concern about falls during daily activities^11^. 

 Despite the established importance of motor and cognitive dysfunctions in PD, the extent of their interdependence and their combined impact on gait remains unclear. Studies have highlighted that cognitive deficits such as diminished executive function and attention, can adversely affect gait performance, increasing the risk of falls and impairing mobility^12^. However, the specific correlations between cognitive assessments, balance metrics, and spatiotemporal gait parameters are not fully understood. Addressing these gaps is critical for advancing rehabilitation strategies, as it may enable the development of targeted interventions that integrate cognitive training and motor rehabilitation to improve functional outcomes in individuals with PD. The interplay between cognitive and motor impairments in PD and their impact on gait stability and independence remains underexplored. More significant cognitive dysfunctions are believed to exacerbate gait impairments and balance instability. Thus, the present study aims to investigate the correlations between cognitive changes, balance deficits, and biomechanical gait parameters in individuals with PD. 

## METHODS

 Data were collected from 22 individuals (17 men; age 64.09±10.51 years; weight 71.42±12.28 kg; height 166.80±7.06 cm; 11 with freezing of gait). The inclusion criteria were individuals with a clinical diagnosis of idiopathic PD, made by a neurologist, who may or may not present freezing of gait. Exclusion criteria were individuals with neurological and orthopedic changes that limited walking. Participants were informed about the objectives, benefits, and risks associated with the study. All participants signed the Free and Informed Consent Form, following the procedures approved by the umbrella project entitled "Effect of dopaminergic medication and disease stages on the gait of patients with Parkinson’s Disease: an open database", approved by the Ethics Committee of the Universidade Federal do ABC in 2019, Certificate of Presentation of Ethical Appreciation (CAAE) number: 21948619.6.0000.5594. 

 The initial assessments consisted of an anamnesis form to collect clinical data, medication, and time since diagnosis of the disease. Then, the following neuropsychological scales were applied:

- Montreal Cognitive Assessment (MoCA): the higher the score, the better the performance^13^;
- Stroop test: the longer the time needed to perform the test, the worse the performance^14^;
- Trail Making Test parts A and B: the longer the time required to perform the test, the worse the performance^14^;

For assessments of motor aspects, the following were carried out:

- UPDRS and Hoehn & Yahr (H&Y), classifying participants into stages I to IV, and the higher the score, the worse the performance (zero score indicates normality)^16^;
- New Freezing of Gait Questionaire (NFOG-Q), indicating the presence of freezing of gait, and the higher the score, the worse the performance^17^;
- Balance Evaluation System Test (BESTest), which has a varied range of balance tests (14 items), including assessments of static, dynamic, and disturbed posture control, in which the higher the score, the better the performance (maximum score indicates normality)^18^;
- International Falls Efficacy Questionnaire (FES-I), where the higher the score, the worse the performance^19^;
- Classification of PD subtype into dominant tremor (TD) and postural instability and gait disturbances (PIGD) was performed according to Stebbins, Goetz 24, using the average of eight scale items to calculate the TD score and five items for the PIGD score.

Individuals whose ratio between the average TD/PIGD scores ≥1.5 were classified as subtype TD, ≤1 as subtype PIGD, and results between >1 and <1.5 were classified as undetermined. In addition, specific motor symptoms related to the TD (UPDRS_TD) and PIGD (UPDRS_PIGD) subtypes were assessed using the average scores of the same items used for classification. 

 The same evaluator performed all clinical evaluations^20^. 

 Participants took part in two experimental sessions, one in the ON state of the medication and the other in the OFF state. To be considered an ON state, participants had taken the dopaminergic medication one hour before starting the session to ensure dosage stabilization. In the OFF state, participants had not taken any PD medication for at least 12 hours at the time of the experiment. The order of sessions was randomized among participants 

 After initial clinical assessments and a 10-minute rest period, participants performed ten attempts at the experimental task. They were instructed to walk at a comfortable speed of 10 m over an electronic walkway. The attempts were made in a row without resting. The evaluator was positioned nearby to guarantee protection and prevent participants from falling during the experimental procedure. 

 In the middle of the walkway, at ground level, there was a pressure system (FDM, Zebris), consisting of two linked electronic walkways. The Zebris system is an electronic walkway that contains pressure-activated sensors embedded in a 60 cm wide x 6 m long mat, which enables the measure of gait parameters in real time by detecting the change in pressure exerted by the participant’s feet when walking on the walkway. The data is automatically transferred to a computer connected to the system to be processed and analyzed. 

 The average of all occurrences of a given variable in the ten trials was used to calculate the variables. The spatiotemporal gait parameters used as variables were:

- Walking speed: average speed, computed as a function of the time used to cover the 6 m walkway distance.
- Cadence: given by the number of steps per minute.
- Average step length: given by the distance from the heel of one foot to the heel of the other foot, in each step, during walking. For analysis, an average was made between the length of the right and the length of the left step.
- Step time: given by the time interval obtained from two successive contacts of the feet with the ground.
- Step width: transverse distance from the center of the heel of one foot to the center of the heel of the other.
- Stride length: distance obtained from two successive contacts of the same foot, measured from the heel.
- Stride time: given by the time interval obtained from two successive contacts of the same foot with the ground.
- Relative support phase time: given by the time stride percentage, considering single and double support, used during the support phase, in which one of the feet is in contact with the ground.
- Double support relative time: given by the percentage of stride time used in the double support phase, in which both feet are in contact with the ground at the same time.
- Swing phase relative time: given by the percentage of stride time used in the swing phase, in which one of the feet is out of contact with the ground, being projected forward to make the next contact.

In statistical analyses, the normality of the dependent variables was verified using the Kolmogorov-Smirnov and Shapiro-Wilk tests. Using Jeffrey’s Amazing Statistics Program (JASP) software, Spearman correlation tests (non-parametric) were performed between the clinical scales and the gait biomechanical variables. Correlations were covariate by disease duration, age, and levodopa in the ON state and disease duration and age in the OFF state of medication. The significance level adopted in all analyses was 5%. 

## RESULTS

 The data collected from 22 individuals were analyzed. These participants were classified between stages I and IV of PD according to the Modified H&Y Scale, with an average score of 2 (range: 1–4). Their cognitive performance, assessed using the MoCA, had an average score of 24 (range: 14–30). 

 The clinical characteristics of the participants are detailed in Table 1. To evaluate the influence of medication on spatiotemporal gait parameters, comparisons were conducted between the ON and OFF medication states, with the results summarized in Table 2. Additionally, the correlations between spatiotemporal gait parameters and clinical scales were analyzed and are presented in Table 3 for the ON state and Table 4 for the OFF state. 

**Table 1:** Average (standard deviation) of participants’ clinical characteristics separately by condition.

Characteristics	ON State	OFF State	P-value
Time of illness (years)	10.32 (5.96)	-	-
LEDD (score)	821.48 (517.25)	-	-
MoCA (score)	22.86 (4.37)	23.36 (4.16)	0.494
FES-I (score)	27.2 (10.6)	34.6 (12.4)	0.001*
Hoehn & Yahr (score)	2.27 (0.70)	2.36 (0.66)	0.346
UPDRS-II Total (score)	4.64 (3.76)	6.68 (3.83)	0.007*
UPDRS-III Total (score)	23.77 (13.24)	26.45 (12.04)	0.172
Mini-BESTest (score)	25.41 (5.23)	24.18 (5.61)	0.059
TUG time (s)	9.90 (3.76)	13.05 (8.38)	0.018*
TUG dual task – time (s)	17.58 (11.81)	27.92 (31.06)	0.085
Stroop-I time (s)	21.15 (9.07)	18.48 (4.95)	0.164
Stroop-II time (s)	23.28 (9.24)	22.90 (7.87)	0.649
Stroop-III time (s)	36.55 (30.75)	31.86 (15.40)	0.390
TMTA time (s)	65.29 (29.78)	62.50 (31.71)	0.848
TMTB time (s)	164.76 (82.94)	160.01 (88.33)	0.614
TMTB/TMTA time (s)	2.72 (1.47)	2.61 (1.17)	0.517
PIGD (score)	0.68 (0.57)	0.93 (0.71)	0.014*
TD (score)	0.59 (0.51)	0.89 (0.69)	0.009*

Abbreviations: FES-I, International Falls Effectiveness Questionnaire; MoCA, Montreal Cognitive Assement Scale; UPDRS-III, Unified Parkinson’s Disease Rating Scale, motor part; UPDRS-II, Unified Parkinson’s Disease Rating Scale, motor aspects of daily life experiences part; Mini-BESTest, Mini-Test of Balance Assessment System; TUG, Time Up and Go Test; Stroop-I,II,III, Stroop test, first, second and third part; TMTA, Trail Making Test, part A; TMTB, Trail Making Test, part B; LEDD, Levadopa Equivalent; PIDG, PD subtype/phenotypic categorization with posture and gait instability; TD, PD subtype/phenotypic categorization with dominant tremor; OFF State, assessment carried out 12 hours after administration of dopaminergic medication; *indicates a significant difference (p<0.05).

**Table 2:** Average (standard deviation) of the spatiotemporal gait parameters separately by condition.

Spatiotemporal Parameters	ON State	OFF State	P-value
Walking speed (m/s)	3.56 (1.02)	3.00 (1.08)	<0.001*
Gait speed variability (%)	7.76 (5.70)	9.08 (5.60)	0.121
Cadence (steps/min.)	54.81 (6.16)	53.52 (8.76)	0.290
Average step length (cm)	53.79 (12.01)	46.19 (15.34)	<0.001*
Step time (s)	0.56 (0.07)	0.59 (0.17)	0.463
Step width (cm)	10.81 (4.32)	11.09 (30.44)	0.726
Stride length (cm)	107.08 (24.12)	92.40 (30.68)	<0.001*
Stride time (s)	1.11 (0.14)	1.17 (0.34)	0.262
Relative swing phase time (%)	34.61 (4.09)	32.45 (7.00)	0.003*
Relative support phase time (%)	65.39 (4.09)	67.55 (7.00)	0.003*
Relative double support time (%)	31.06 (7.98)	35.62 (15.71)	0.010*

Abbreviations: ON State, assessment carried out 1 hour after administration of dopaminergic medication; OFF State, assessment carried out 12 hours after administration of dopaminergic medication.

**Table 3:** Correlations between clinical scales and gait in the ON State.

		H_Y	FES-I	UPDRS-II	UPDRS-III	MoCA	MiniBest	TUG – Time (s)	TUG Dual Task – Time (s)	STROOP-III - Time (s)	TMTB-A	PIGD	TD
**Step length**	Spearman’s rho	-0.43	-0.48*	-0.25	-0.33	0.202	0.508*	-0.44	-0.15	-0.12	-0.49*	-0.42	-0.05
	p-value	0.067	0.039	0.311	0.167	0.407	0.026	0.059	0.53	0.621	0.035	0.075	0.855
Stride length	Spearman’s rho	-0.42	-0.42	-0.24	-0.313	0.144	0.479*	-0.4	-0.15	-0.09	-0.46*	-0.39	-0.1
	p-value	0.076	0.074	0.327	0.192	0.555	0.038	0.087	0.544	0.722	0.047	0.101	0.697
Step width	Spearman’s rho	0.542*	0.565*	0.507*	0.219	0.166	-0.68**	0.189	0.158	0.377	0.285	0.464*	-0.03
	p-value	0.017	0.012	0.027	0.367	0.497	0.001	0.439	0.519	0.112	0.238	0.046	0.905
Cadence	Spearman’s rho	-0.49*	-0.52*	-0.53*	-0.253	0.268	0.698***	-0.52*	-0.65**	-0.49*	-0.48*	-0.63**	0.198
	p-value	0.032	0.022	0.02	0.295	0.267	<0.001	0.022	0.003	0.033	0.036	0.004	0.416
Step time	Spearman’s rho	0.445	0.387	0.487*	0.217	-0.185	-0.61**	0.454	0.654**	0.406	0.414	0.538*	-0.11
	p-value	0.056	0.102	0.034	0.372	0.449	0.006	0.051	0.002	0.085	0.078	0.018	0.668
Stride time	Spearman’s rho	0.493*	0.512*	0.533*	0.261	-0.271	-0.7***	0.527*	0.646**	0.476*	0.48*	0.625**	-0.19
	p-value	0.032	0.025	0.019	0.28	0.261	<0.001	0.02	0.003	0.039	0.037	0.004	0.446
**Gait Speed**	Spearman’s rho	-0.48*	-0.6**	-0.34	-0.392	0.35	0.658**	-0.54*	-0.34	-0.29	-0.65**	-0.56*	0.119
	p-value	0.036	0.007	0.149	0.097	0.141	0.002	0.018	0.154	0.221	0.003	0.013	0.629
Swing phase	Spearman’s rho	-0.66**	-0.75**	-0.5*	-0.198	-0.051	0.559*	-0.4	-0.55*	-0.65**	-0.41	-0.65**	-0.05
	p-value	0.002	<0.001	0.03	0.417	0.836	0.013	0.092	0.016	0.003	0.085	0.003	0.83
Stance phase	Spearman’s rho	0.66**	0.745***	0.497*	0.198	0.051	-0.56*	0.397	0.546*	0.648**	0.406	0.652**	0.053
	p-value	0.002	<0.001	0.03	0.417	0.836	0.013	0.092	0.016	0.003	0.085	0.003	0.83
Double support phase	Spearman’s rho	0.657**	0.649**	0.497*	0.185	0.146	-0.51*	0.37	0.551*	0.569*	0.313	0.612**	0.179
	p-value	0.002	0.003	0.03	0.448	0.55	0.025	0.119	0.014	0.011	0.193	0.005	0.464

* p<0.05;

** p<0.01;

*** p<0.001.

Conditioned on the variables: age, levadopa equivalent, duration of the disease.

**Table 4:** Correlations between clinical scales and gait in the OFF State.

		H_Y	FES-I	UPDRS-II	UPDRS-III	MoCA	MiniBest	TUG – Time (s)	TUG Dual Task – Time (s)	STROOP-III - Time (s)	TMTB-A	PIGD	TD
**Step length**	Spearman’s rho	-0.59**	-0.52*	-0.57**	-0.15	-0.09	0.326	-0.56**	-0.59**	-0.04	-0.056	-0.66**	0.29
	p-value	0.007	0.019	0.009	0.536	0.706	0.161	0.01	0.006	0.877	0.82	0.001	0.214
Stride length	Spearman’s rho	-0.59**	-0.52*	-0.57**	-0.15	-0.09	0.326	-0.56**	-0.59**	-0.04	-0.056	-0.66**	0.29
	p-value	0.007	0.019	0.009	0.536	0.706	0.161	0.01	0.006	0.877	0.82	0.001	0.214
Step width	Spearman’s rho	0.568**	0.32	0.1	0.489*	-0.054	-0.6**	0.31	0.364	0.281	0.204	0.108	-0.1
	p-value	0.009	0.169	0.674	0.029	0.821	0.005	0.184	0.114	0.229	0.401	0.652	0.677
Cadence	Spearman’s rho	-0.3	-0.3	-0.18	-0.37	-0.056	0.246	-0.16	-0.09	-0.35	-0.428	-0.2	-0.04
	p-value	0.204	0.207	0.443	0.11	0.816	0.296	0.507	0.709	0.132	0.067	0.398	0.857
Step time	Spearman’s rho	0.313	0.31	0.192	0.354	0.075	-0.25	0.177	0.117	0.351	0.45	0.211	0.026
	p-value	0.178	0.184	0.418	0.126	0.752	0.297	0.454	0.622	0.129	0.053	0.373	0.912
Stride time	Spearman’s rho	0.303	0.301	0.19	0.365	0.065	-0.25	0.161	0.091	0.342	0.415	0.206	0.045
	p-value	0.195	0.197	0.423	0.114	0.786	0.282	0.498	0.703	0.14	0.077	0.385	0.849
**Gait Speed**	Spearman’s rho	-0.66**	-0.6**	-0.68**	-0.25	-0.138	0.415	-0.6**	-0.62**	-0.16	-0.243	-0.73***	0.181
	p-value	0.002	0.006	0.001	0.284	0.563	0.069	0.005	0.003	0.489	0.316	<0.001	0.444
Swing phase	Spearman’s rho	-0.51*	-0.74***	-0.67**	-0.25	0.073	0.345	-0.48*	-0.56*	-0.57**	-0.335	-0.61**	0.215
	p-value	0.022	<0.001	0.001	0.289	0.761	0.136	0.032	0.011	0.008	0.16	0.004	0.363
Stance phase	Spearman’s rho	-0.51*	0.737***	0.666**	0.249	-0.073	-0.35	0.481*	0.557*	0.574**	0.335	0.612**	-0.22
	p-value	0.022	<0.001	0.001	0.289	0.761	0.136	0.032	0.011	0.008	0.16	0.004	0.363
Double support phase	Spearman’s rho	0.496*	0.722***	0.665**	0.229	-0.064	-0.34	0.469*	0.56*	0.567**	0.335	0.594**	-0.19
	p-value	0.026	<0.001	0.001	0.331	0.789	0.146	0.037	0.01	0.009	0.16	0.006	0.413

* p<0.05;

** p<0.01;

*** p<0.001.

Conditioned on the variables: age, duration of the disease.

## DISCUSSION

 Correlating the clinical cognitive and motor scales with various gait parameters assessed in this study revealed significant associations. In the ON medication state, a strong relationship was observed between the risk of falls, as evaluated by the FES-I test, and gait parameters, indicating that a more stable and effective gait corresponds to a reduced risk of falls. 

 Furthermore, inverse relationships were identified between the Stroop Test, TUG, and TMTB-A, which assess inhibitory control, functional mobility, and cognitive flexibility. These findings show that longer test completion times correlate with poorer performance, including reduced cadence and stride time. In contrast, a wider stride size was associated with better outcomes in these tests. Similarly, the Mini-BESTest, which evaluates dynamic balance, showed direct proportionality with multiple gait parameters, reinforcing the direct relationship between balance and gait. 

 Given the complexity of cognitive impairments in PD, gait has emerged as a promising, low-cost, and non-invasive clinical biomarker for predicting cognitive decline and dementia^21^. Studies suggest that understanding the underlying mechanisms of gait dysfunction in PD—characterized by postural instability, reduced stride length, and loss of movement dissociation between the trunk and arms—could help address the increased risk of falls associated with the disease^22^. Balance and gait disturbances likely result from abnormal projections from the basal ganglia to midbrain locomotor regions and dysfunctional dopamine modulation in direct cortical pathways, which are consequences of dopaminergic neuronal degeneration in the substantia nigra^2^. 

 Supporting these findings, Lord et al. (2014) demonstrated that executive dysfunctions in individuals with PD are associated with decreased gait speed and swing phase duration, alongside increased variability in gait speed and step timing^23^. Poor performance on the TMT-A and TMT-B, which measure motor speed, inhibitory control, and cognitive flexibility, was also linked to worse functional mobility outcomes, consistent with the present study’s findings in the ON state^24^. 

 The MoCA test, widely used for cognitive assessment, has been shown by Morris et al.^25^ to predict attention decline in relation to gait parameters. However, its ability to predict attention fluctuation and visual memory was limited. Interestingly, this study did not find significant correlations between MoCA scores and spatiotemporal gait parameters, which might be explained by some participants’ extremely low MoCA scores. These results may reflect socioeconomic disparities and educational inequities in the Brazilian population^25^. Bezerra^26^ suggests that employing multiple cognitive screening tools could enhance assessment accuracy in such contexts. 

 Postural balance depends on the dynamic interaction of sensory, cognitive, and motor systems. In PD, cognitive dysfunctions affect quality of life and impair motor performance, leading to decreased gait speed^22,27^. Non-motor dysfunctions, such as those arising from neuronal loss in the locus coeruleus (affecting mood), the Meynert’s nuclei (affecting attention and memory), and the raphe nuclei (affecting sleep and emotional regulation), contribute to the disease’s complexity^3^. 

 Bloem et al.^28^ evaluated various motor and classification scales for PD and recommended the PIGD score from the UPDRS, the Mini-BESTest, and the TUG as effective tools for assessment and treatment, all of which were utilized in the present study. Similarly, Kluger et al.^29^ reported significant correlations between UPDRS scores and resistance, turning ability, and attention, while the TUG test was linked to gait speed, stride length, turning performance, and attentional control. 

 In the OFF medication state, the UPDRS-II, which evaluates daily living activities, showed significantly higher scores, indicating worse performance. However, no significant differences were observed in UPDRS-III scores, which assess motor aspects, except for a direct proportionality between UPDRS-III and step width in the OFF state (Table 4). This unexpected finding of poor levodopa responsiveness might be associated with advanced age, advanced disease, or PD subtypes of poorer prognosis^30^. As gait showed significant improvements, the levodopa dosage was probably insufficient for a "robust" medication status, perhaps as habitual dosages were used. 

 The present study also confirmed the well-known phenomenon that individuals with PD walk slower when performing dual tasks, as evidenced by the longer completion times for the TUG test under dual-task conditions^31^. Neurodegenerative changes and cholinergic dysfunctions in PD are linked to reduced attentional capacity, making gait regulation more susceptible to environmental influences^32^. Basal ganglia dysfunction further amplifies the reliance on cognitive processes for gait control in PD, highlighting the importance of attention in gait performance^33^. 

 Thus, attention plays a central role in the gait of individuals with PD^34^. Stuart^35^ emphasized the need for precise and independent evaluation of cognitive assessments and their interaction with gait parameters. Although this study observed longer completion times for selective attention (Stroop Test) and visual attention (TMT) tasks in the ON state, these results align with Iansek et al.^33^, who reported that dopaminergic medication may impair task-specific focus and induce dyskinesia. 

 Consistent with previous findings, this study observed that gait rhythm parameters appear resistant to levodopa, while spatial parameters are more sensitive to the medication^36^. Speed, step length, and stride length increased in the ON state, while step and stride times showed no significant differences, corroborating Mondal’s (2019) findings^36^. Fukuchi et al.^37^ suggested that to compare gait parameters in groups with different self-selected speeds, as occurs in the ON-OFF phenomenon, it is necessary to reduce the impact of speed in other variables, using mathematical models to adjust the results according to gait speed. 

 Ultimately, the ability to create gait improvement strategies in PD depends on leveraging cognitive capacities, such as attention and mental imagery^33^. Addressing cognitive impairments alongside motor deficits in rehabilitation may enhance gait safety and effectiveness. Moreover, optimizing medication regimens is crucial to minimizing fluctuations, dyskinesia, and other adverse effects^38^. 

 This study highlights how atypical gait patterns in PD are intricately linked to cognitive impairments. The characteristic festinating gait—marked by a flexed posture, reduced dissociation of pelvic and shoulder girdle movements, and short, shuffling steps—amplifies fall risk. Thus, interventions should integrate cognitive rehabilitation, focusing on attention and memory training alongside motor control and muscle strength optimization, to achieve safer and more effective gait outcomes. 

## Data Availability

The datasets generated and/or analyzed during the current study are available from the corresponding author upon reasonable request.
